# Functional and Structural Connectivity Between the Left Dorsolateral Prefrontal Cortex and Insula Could Predict the Antidepressant Effects of Repetitive Transcranial Magnetic Stimulation

**DOI:** 10.3389/fnins.2021.645936

**Published:** 2021-03-26

**Authors:** Yixiao Fu, Zhiliang Long, Qinghua Luo, Zhen Xu, Yisijia Xiang, Wanyi Du, Yuanyuan Cao, Xiaoli Cheng, Lian Du

**Affiliations:** ^1^Department of Psychiatry, The First Affiliated Hospital of Chongqing Medical University, Chongqing, China; ^2^Sleep and NeuroImaging Center, Faculty of Psychology, Southwest University, Chongqing, China

**Keywords:** repetitive transcranial magnetic stimulation, resting-state functional magnetic resonance imaging, diffusion tensor imaging, dorsolateral prefrontal cortex, insula

## Abstract

**Background:**

The efficacy of repetitive transcranial magnetic stimulation (rTMS) in depression is nonuniform across patients. This study aims to determine whether baseline neuroimaging characters can provide a pretreatment predictive effect for rTMS.

**Methods:**

Twenty-seven treatment-naive patients with major depressive disorder (MDD) were enrolled and scanned with resting-state functional magnetic resonance imaging (fMRI) and diffusion tensor imaging. Clinical symptoms were assessed pre- and post-rTMS. Functional and structural connectivity between the left dorsolateral prefrontal cortex (DLPFC) and bilateral insula were measured, and the connectivity strength in each modality was then correlated to the clinical efficacy of rTMS.

**Results:**

When the coordinates of left DLPFC were located as a node in the central executive network, the clinical efficacy of rTMS was significantly correlated with the functional connectivity strength between left DLPFC and bilateral insula (left insula: *r* = 0.66; right insula: *r* = 0.65). The structural connectivity strength between the left DLPFC and left insular cortex also had a significantly positive correlation with symptom improvement (*r*_*s*_ = 0.458).

**Conclusion:**

This study provides implications that rTMS might act more effectively when the pretreatment functional and structural connectivity between the insula and left DLPFC is stronger.

## Introduction

The pathophysiology of depression involves dysfunction in both limbic and cortical regions (Mayberg, 2007). Among these regions, the left DLPFC (Koenigs and Grafman, 2009) and insula (Sliz and Hayley, 2012) have been extensively studied given their high rate of involvement in depression-related abnormalities. The DLPFC is a key region of executive control network (CEN), mediating cognitive functions. Functional imaging studies have revealed hypoactivity in the left DLPFC during the progression of depression and hyperactivity during the recovery phase (Koenigs and Grafman, 2009). Lesion studies in patients have shown that relative to nonfrontal lesions, bilateral dorsal PFC lesions were associated with substantially higher levels of depression (Koenigs and Grafman, 2009). The insula has distributed connectivity with the prefrontal, temporal, parietal, and limbic regions (Sliz and Hayley, 2012), which has been putatively implicated as an integration center between the external environment and internal processing and regulating regions in the brain (Sliz and Hayley, 2012). Tracer studies in primates have shown direct white matter connections between the anterior insula and prefrontal regions (Mesulam and Mufson, 1982). The anterior insula has been reported to be associated with the cognitive–affective dimension (Peltz et al., 2011) and further reported to be a part of a “salience network” (SN) (Sridharan et al., 2008). A growing body of literature has identified insula dysfunction in adolescents at-risk for (Gotlib et al., 2010) and with depression (Tahmasian et al., 2013; Jacobs et al., 2016; Yin et al., 2018). Changes in insula activity have been thought to be associated with antidepressant treatments (Fu et al., 2013).

Some previous findings have shown aberrant connectivity between insular and dorsal lateral frontal regions connectivity in sub-threshold depression patients (Hwang et al., 2015), as well as between SN and CEN in depression (Manoliu et al., 2014). Sevdalina et al. further showed that patients had significant reduced strength of the connection from the anterior insula to the DLPFC in depression (Kandilarova et al., 2018). Moreover, Hyett et al. (2015) found that melancholic patients demonstrated weaker effective connectivity between the right frontoparietal and insula networks. This suggests the possibility that a disrupted connectivity between the DLPFC and the insula in depression.

Repetitive transcranial magnetic stimulation (rTMS) is regarded as a promising treatment option for depression (Hallett, 2007), has been approved by the US Food and Drug Administration (FDA), and is covered by many public and private insurers in the USA and other countries (Perera et al., 2016). However, rTMS treatment for depression usually takes 4-6 weeks, and only a portion of patients can benefit from rTMS, while others could not (O’Reardon et al., 2007). Therefore, it is of critical importance to obtain a measurement that can provide predictive value on the treatment efficacy of rTMS in depression. The left DLPFC was one of regions targeted most often in treating depression with rTMS. Notably, TMS can not only directly affect regions at the cortical surface but also propagate beyond the site of stimulation, impacting a distributed network of brain regions (Ferreri et al., 2011; Fox et al., 2012b) including the insula (Kito et al., 2011). Thus, we hypothesized that rTMS took effects through the connectivity between left DLPFC (directly stimulated region) and insula. To elucidate these issues, the present study used resting-state functional connectivity magnetic resonance imaging (MRI) (Van Dijk et al., 2010) and diffusion tensor imaging (DTI) to determine whether resting-state functional and structural connectivity between the left DLPFC and bilateral insula can provide a pretreatment predictive measure for the antidepressant effect of rTMS.

## Materials and Methods

### Participants

A total of 27 right-handed outpatients (18 females, 9 males; age: 40 ± 13.8 years), with a single episode or recurrent depressive episodes and who were medication-free (not treated in the previous month and had been previously medicated for less than a week) were recruited for this study. The diagnosis of MDD was confirmed by the Structured Clinical Interview for the Diagnostic and Statistical Manual of Mental Disorders IV (SCID-I/P, Chinese version). Patients were excluded if they had a history of current or past psychotic disorders, alcohol or drug abuse, any current clinically significant neurological disorder or other serious physical diseases, morphological abnormalities in the brain, less than 18 years of age, and any electronic or metal implants. The therapeutic schedule for the MDD patients was decided by their clinicians; we only observed and recorded the relevant situations. At the beginning of this study, patients were screened by the researchers to assess if they met the above inclusion/exclusion criteria. All the patients were assessed for clinical severity using the 17-item Hamilton Depression Scale (HAMD) both at baseline and at the end of rTMS treatment. This study was reviewed and approved by the Local Medical Ethics Committee of the First Affiliated Hospital of Chongqing Medical University. All participants provided written informed consent before participating in this work.

### rTMS Protocol

rTMS was delivered in sessions by a YRD CCY-I magnetic simulator (YIRUIDE Inc., Wuhan, China). The patients received a total of 10 sessions of rTMS (five sessions per week for 2 weeks). The stimulation parameters were within the safe range requirements (George and Belmaker, 2007): 100% magnetic field strength relative to the patient’s observed resting motor threshold, at 10 pulses per second for 3 s, with an interval of 21 s. Treatment sessions lasted for 20 min (50 trains) and consisted of 1,500 pulses. The stimulation position for the left DLPFC was located using the “5 cm” method (Johnson et al., 2013) (with the left DLPFC target as a point located 5 cm in front of the “hand motor hotspot” in the parasaggital plane pointing anteriorwards).

### MRI Scan Acquisition

The 27 patients were scanned by MRI at baseline. Data were acquired on a 3.0 Tesla MRI system (GE Medical Systems, Waukesha, WI, United States) at the First Affiliated Hospital of Chongqing Medical University. The participants were asked to remain motionless, keep their eyes closed, and not think of anything. Functional images were obtained by using an echo-planar imaging sequence with the following parameters: TR/TE = 2000/30 ms, 33 axial slices, field of view = 240 × 240 mm^2^, matrix size = 64 × 64, voxel size = 3.75 × 3.75 × 5 mm^3^, flip angle = 90°, and a total of 240 volumes. The T1 structural images were acquired using the following parameters: TR/TE = 8.35/3.27 ms, slice thickness = 1 mm, flip angle = 12°, matrix size = 256 × 256, and 156 sagittal slices. The parameters for the DTI scanning were as follows: TR/TE = 15000/88.3 ms, slice thickness = 2 mm, flip angle = 90°, 56 axial slices, matrix size = 256 × 256, 30 noncollinear diffusion weighting gradient directions [b = 1000 s/mm^2^], and four additional images without diffusion weighting [b = 0 s/mm^2^].

### Functional Data Preprocessing and Functional Connectivity Analysis

The preprocessing of the resting-state MRI data was performed using the SPM12 software toolbox^1^. The first 10 time points were discarded due to the adaptation of the participants to the scanning environment and magnetization stabilization. The images were then corrected for the time-delay between slices and the motion movement between volumes. The participants with their x, y, or z directions larger than 3 mm or rotation around each axis larger than 3° were excluded. All subjects met the criterion. The resulting images were normalized by using a unified segmentation of anatomical images and resampled into a 3 × 3 × 3 mm^3^ voxel size. A multiple regression model was employed to remove the effect of covariates of no interest, including 24 motion parameters, mean white matter signal and mean CSF signal. The resulting images were finally linearly detrended and filtered in the range of 0.01 to 0.08 Hz.

Given that resting-state functional connectivity (FC) is sensitive to minor head movements (Power et al., 2012, 2014, 2015; Satterthwaite et al., 2013), we computed the framewise displacement (FD) (Power et al., 2012) at time point *i*, which is defined as follows:

$$FD_{i}=\left|\Delta d_{ix}\right|+\left|\Delta d_{iy}\right|+\left|\Delta d_{iz}\right|+r\left|\Delta \alpha_{i}\right|+r\left|\Delta \beta_{i}\right|+r\left|\Delta \gamma_{i}\right|$$

where the Δ*d*_*ix*_ = *d*_(*i*−1)*x*_−*d*_*ix*_ and is similar for the other parameters Δ*d*_*iy*_, Δ*d*_*iz*_, Δα_*i*_, Δβ_*i*_, and Δγ_*i*_. The radius *r* is 50 mm, which is the approximate mean distance from the cortex to the center of the head. The “bad” time points as well as their 1-back and 2-forward time points were removed from the time series by employing a “scrubbing” method (Power et al., 2012) with an FD threshold of 0.5 mm. The participants retaining more than 80% of the original signals after scrubbing were included in further analyses.

The spherical regions of interest (ROI) of the left DLPFC were obtained with the center of -36 27 29 and radius of 8 mm (de Kwaasteniet et al., 2015), according to the coordinates of a node in CEN, and multiplied by a mask of the automated anatomical labeling (AAL) atlas (Tzourio-Mazoyer et al., 2002) to exclude none gray matter voxels. The ROIs of the bilateral insular cortex were selected based on the AAL atlas.

Seed-based functional connectivity (FC) analysis was conducted between the averaged time course of the left DLPFC and the time series of all voxels across the insular cortex for each subject by using Pearson’s correlations. The correlation values were subsequently *r*-to-*z* transformed. A two-tailed one-sample *t*-test was then employed to test whether the FC value was different from zero. Voxels with *p* < 0.05 were saved as a mask for the following multiple regression analysis.

Considering the small sample size in the current study, multiple regression analysis was conducted between FC maps and the HAMD score reductions by employing Statistical Non-Parametric Mapping (SNPM version 13)^2^ with 5000 permutations (Nichols and Holmes, 2001). The multiple comparisons were corrected by using the cluster-size-based familywise error of *p* < 0.05 with a cluster-forming threshold of *p* < 0.005.

It has been suggested that resting-state functional connectivity is sensitive to head movement. So we computed mean FD for each subject, and included it as a covariate, in order to see if head movement has influence on the statistical results.

Considering the different coordinates of the left DLPFC according to different methods, we further used another two methods to locate the left DLPFC, the center of BA9 (−36 39 43) and the center of BA46 (−44 40 29), and then separately repeated the above steps.

### Diffusion Tensor Image Analysis

The preprocessing of DTI images was carried out using the following procedures. Briefly, each individual T1-weighted image was first coregistered to the B0 image in the native diffusion space by using a linear transformation. The coregistered structural images were then mapped to the MNI T1-template. The derived transformation parameters were inverted and used to warp the ROIs of the left DLPFC and bilateral insular cortex from the MNI space to the native diffusion space. For the structural connectivity analysis, we selected BA9 plus BA46 as the ROI of the left DLPFC. These procedures were conducted using SPM12 software.

For each subject, diffusion-weighted images were corrected for the eddy-current-induced distortions and head movements using FSL^3^. Diffusion tensor models were estimated by the linear least-squares fitting method at each voxel using the Diffusion Toolkit (Wang et al., 2007). Whole-brain fiber tracking was performed in native diffusion space by employing the Fiber Assignment by Continuous Tracking (FACT) algorithm. All the tracks in the data were computed by seeding voxels with fractional anisotropy (FA) larger than 0.2 (Mori et al., 2002). Path tracing continued until either a voxel with FA less than 0.2 was reached or the angle between the current and the previous path segment exceeded 45 degrees (Shu et al., 2011).

We calculated the mean FA values of connected fibers between the left DLPFC and insular cortex. The FA value is an important index to evaluate fiber integrity (Beaulieu, 2002) and can detect local subtle brain lesions (Lim and Helpern, 2002). Furthermore, nonparametric Spearman correlation analysis was performed to investigate the relationship between the nonzero FA values and the corresponding HAMD score reductions. A statistical level of p < 0.05 was considered significant.

## Results

### Clinical Outcomes

**FIGURE 1:**
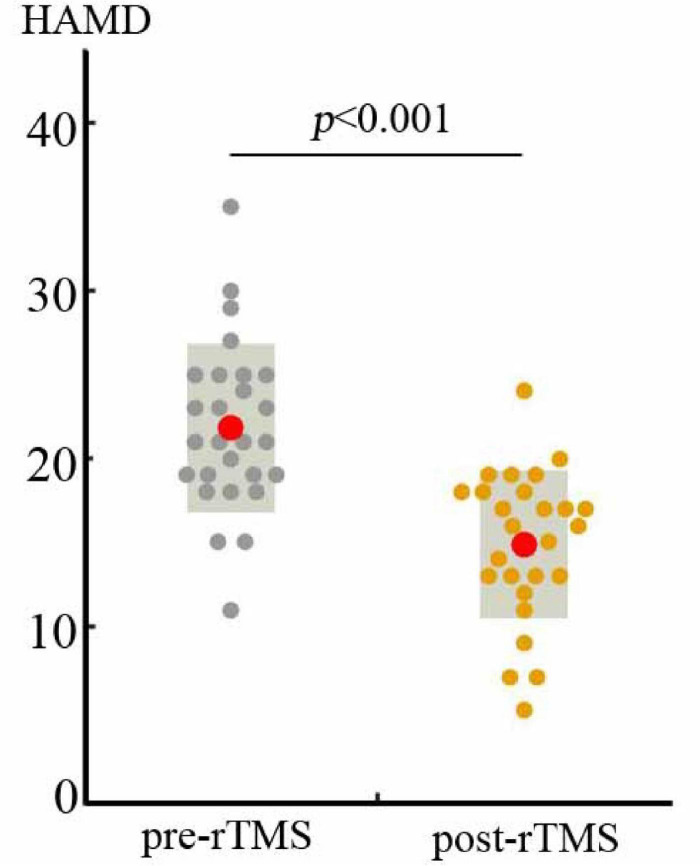
Two weeks of rTMS induced significant decreases in HAMD scores (*t* = 5.97, *p* < 0.001).

The demographic and clinical features of the MDD patients are summarized in Table 1. After 2 weeks of rTMS treatment, the total scores on the HAMD were significantly decreased (*t* = 5.97, *p* < 0.001; Figure 1), with the HAMD reductive rate of 0.29 ± 0.24 (mean ± std).

**TABLE 1:** Demographics and clinical characters of MDD patients.

Demographics	MDD (*n* = 27)	*p*-value	*T*-value
Age (years)	40.04 ± 13.79	/	/
Sex (male/female)	9/18	/	/
Episode (first/recurrent)	15/12	/	/
Education (years)	12.00 ± 3.95	/	/
Age of onset (years)	34.44 ± 13.33	/	/
HAMD scores: Pre-rTMS	21.81 ± 5.04	*p* < 0.0001^a^	5.97
Post-rTMS	14.89 ± 4.41		
HAMD reduction rate	0.29 ± 0.24		

*The values are illustrated as mean ± SD.MDD, major depressive disorder;HAMD, Hamilton Depression Scale.^a^ paired t-test.*

### Correlation of Functional Connectivity at Baseline With Clinical Efficacy

**FIGURE 2:**
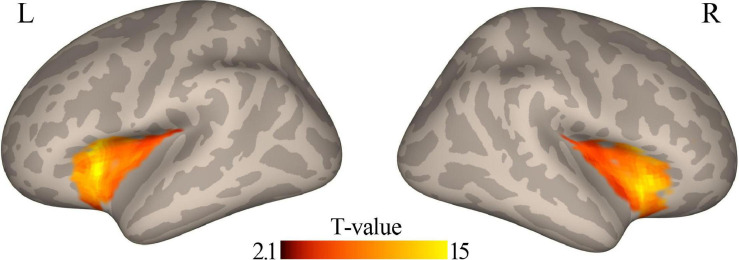
Positive functional connectivity between the left DLPFC and bilateral insular cortex was observed using a one-sample *t*-test (*p* < 0.05). DLPFC, dorsal lateral prefrontal cortex.

**FIGURE 3:**
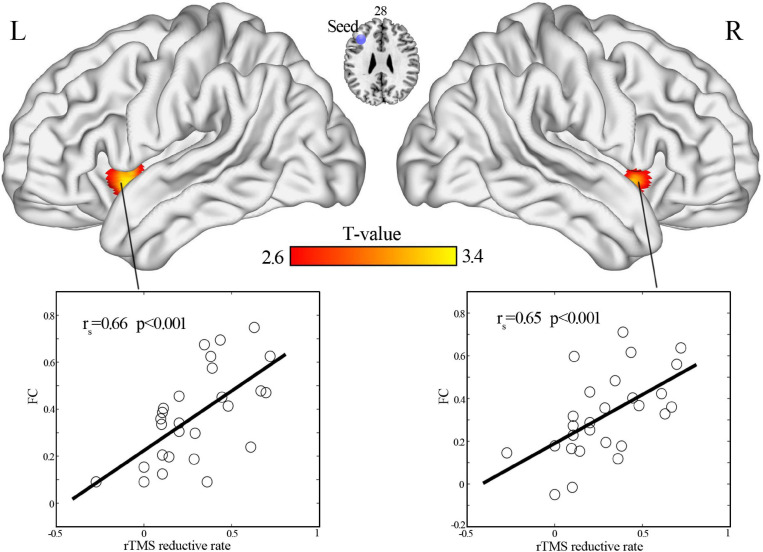
Positive correlation of HAMD scores reduction with functional connectivity between the left DLPFC and bilateral anterior insular cortex (cluster-wise and family-wise error correction with voxel *p* < 0.005 and cluster *p* < 0.05). DLPFC, dorsal lateral prefrontal cortex.

When the coordinates of the left DLPFC were located according to the coordinates of a node in the CEN, a one-sample *t*-test revealed that the left DLPFC had positive functional connectivity with the bilateral insular cortex (Figure 2). In addition, functional connectivity between the left DLPFC and bilateral anterior insular cortex was significantly correlated (*r* = 0.66, *p* < 0.001, for left insula; *r* = 0.65, *p* < 0.001, for the right insula; Figure 3).

Whether included mean FD as a covariate or not in the statistical analysis, we both found significantly positive correlation between left DLPFC-left insula FC and clinical efficacy of rTMS.

When the coordinates of the left DLPFC were located at the center of BA9 and BA46, we did not observe any significant correlations between the connectivity of the left DLPFC with the insula and the clinical efficacy of rTMS (Supplementary Figures 1, 2).

### Correlation of Structural Connectivity at Baseline With Clinical Efficacy

**FIGURE 4:**
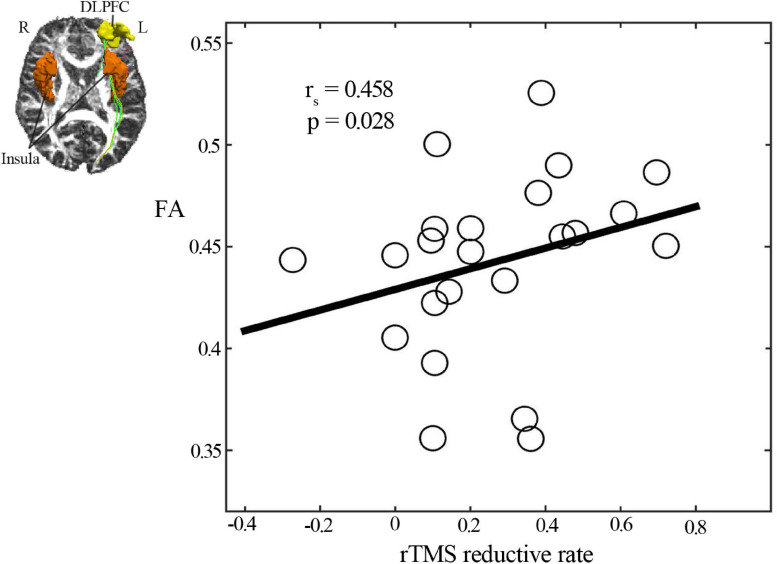
Spearman correlation analysis showed a positive correlation of clinical efficacy with the mean FA value of structural connectivity between the left DLPFC and left insular cortex. DLPFC, dorsal lateral prefrontal cortex; FA, fractional anisotropy.

We observed that fiber tracks existed only between the left DLPFC and left insula but not between the left DLPFC and right insula. Analysis of the diffusion tensor images revealed that the mean FA values of structural connectivity between the left DLPFC and left insula cortex had a significantly positive correlation with the rTMS-induced HAMD score reductions (*r*_*s*_ = 0.458, p = 0.028) (Figure 4).

## Discussion

In the current article, we used a connectivity analysis to gain insight into why rTMS using the same ‘5 cm method’ to locate the left DLPFC on the surface of the skull has different clinical effects in depressed patients. We identified a positive correlation between the clinical efficacy of rTMS and functional connectivity (left DLPFC–bilateral insula) in the pre-rTMS state as well as structural connectivity (left DLPFC–left insula). This makes it plausible that different levels of connectivity between the left DLPFC and insula across patients can predict the different effect sizes of rTMS. Interestingly, the predictive effect only occurs when the seed of the left DLPFC was selected as a node in the CEN, rather than the traditional BA9 or BA46. We first combined a functional and structural connectivity approach to illustrate the correlations of the superficial region (left DLPFC) with the deeper brain areas (insula) and found that their connectivity strength could predict the antidepressant effects of rTMS, which suggests that frontal-insular connectivity plays an important role on the predictive mechanism of rTMS in MDD.

The possible mechanisms for the differential effects of rTMS on depression warrant discussion. We examined the predictive neuroimaging mechanisms of rTMS with respect to both functional and structural connectivity of the superficial left DLPFC with deep brain areas that correlate to clinical efficacy. Fox et al. (2012a) found that the antidepressant efficacy of different left DLPFC TMS sites was related to the anticorrelation extent of each site with the subgenual cingulate, but the measures of clinical efficacy in that article were based on previously published data. Moreover, one idea about the mechanisms of rTMS that have been aggressively pursued was the propagation of TMS effects through anatomical connections to deeper limbic regions (Padberg and George, 2009). However, the anticorrelation between subgenual ACC and DLPFC is thought unlikely to be the result of direct inhibitory connections. A growing number of cross-sectional studies have used DTI to investigate the white matter (WM) microstructure in patients with MDD. Despite different methodological approaches, most investigations have reported MDD-related reductions in fractional anisotropy (FA), which is used as an index of WM integrity. A number of previous reviews have pointed to smaller FA values in WM regions associated with frontal, limbic and striatal lobes in patients with MDD, which meant altered regional patterns of WM structural connectivity in those areas (Korgaonkar et al., 2011; Cole et al., 2012). Electroconvulsive treatment (ECT) was thought to modulate the WM microstructure in pathways connecting the frontal and limbic areas in patients with MDD and relate to its therapeutic response (Lyden et al., 2014). Thus, this study combined resting-state fMRI and DTI to calculate the functional correlations and FA values and then explored whether functional and structural connectivity between left DLPFC and insula predicted the clinical efficacy of rTMS.

The current study found that insula (mainly anterior insula) and DLPFC are intrinsically positively correlated in the resting state. There are several implications of this result. First, primary hypoactivity in the left DLPFC might result in secondary hypoactivity of the insula. Second, focal excitation/inhibition in one region could be expected to enhance/suppress activity in the other region. Third, the stronger the correlation between the activities of the two regions, the more likely that rTMS will be fully effective. Lastly, the DTI analysis showed that there were fiber connections between the insula and left DLPFC. This supports the feasibility that rTMS could affect the function of the left insula following direct stimulation of the left DLPFC through their fiber connections.

Moreover, the functional connectivity between the left DLPFC and insula could predict the antidepressant effect of rTMS only when the coordinate of left DLPFC was selected in the node of CEN. If we used other methods, such as the center of BA9 or BA46 as the seed site, the prediction effect was not significant. This emphasized the importance of CEN in the action of rTMS, which could be understood and explained by previous studies about large-scale networks in MDD. Recent studies have shown that depression is characterized by abnormal functional integration of brain networks at rest (Alexopoulos et al., 2012; Liao et al., 2018), mainly including the following three neural networks: the DMN, CEN, and SN (Seeley et al., 2007; Alexopoulos et al., 2012; Kaiser et al., 2015; Liao et al., 2018). In addition, previous study showed that functional connectivity patterns of brain regions between and within networks appeared to play an important role in identifying a favorable response for a drug treatment for MDD (Liston et al., 2014). These neural circuit impairments in depression are involved in the processing of both positive and negative emotional information, attribution of salience, and both cognitive and emotional control (Kaiser et al., 2015). The CEN, which plays a key role in executive function and emotion regulation, includes the DLPFC and lateral posterior parietal regions (Seeley et al., 2007). Meta-analyses have noted consistent patterns of resting-state hypoconnectivity within the executive control network for MDD (Kaiser et al., 2015). The SN is involved in detecting, integrating and processing internal and external salient information, and it includes the dorsal anterior cingulate cortex (dACC), anterior insula, amygdala, and ventral striatum (Seeley et al., 2007). The left DLPFC and anterior insula are the key nodes of the CEN and SN, respectively. To some extent, the strength of connectivity between the left DLPFC and insula reflects the degree of correlation of the CEN and SN. Thus, we speculate the reason why more relevancy predicted better antidepressant effect might be that, rTMS adjusts the function of CEN through stimulating left DLPFC, and then affects the function of SN through connectivity between left DLPFC and insula, and might also affect DMN through the connectivity between CEN and DMN as well as between SN and DMN. The antidepressants effects might be produced through the modulation of the function of the above networks. However, these are only hypotheses and further research is needed to verify the alteration of brain networks induced by rTMS. In summary, the current findings suggest that the antidepressant effect of rTMS might be optimized when the placement location of left DLPFC is according to the CEN. However, the position is lateral or medial, anterior or posterior might not be so important.

While the above discussion focused on the insula and the DLPFC, sgACC is an area of interest in the field of rTMS in depression. Liston et al. (2014) pointed out that rTMS acted by reducing sgACC-to-DMN connectivity and inducing anticorrelated connectivity between the DLPFC and DMN. Fox et al. (2012a) implicated left DLPFC-sgACC functional connectivity in the action of rTMS. However, one of our previous studies in depression found that compared to those not improved, early improvers did not exhibit significant differences in left DLPFC–sgACC functional connectivity strength (Du et al., 2018). Therefore, we speculate that the strength of the connection between left DLPFC and insula might also be a predictor of the rTMS efficacy.

### Limitations

The current work is limited in several respects, and these limitations suggest important avenues for future research. First, the sample size is small. Only 27 patients were included in the analysis. Second, we failed to detect the effect of a sham control group versus active rTMS, which would have made it more convincing that these neuroimaging features could predict the clinical efficacy of rTMS.

## Conclusion

This study explored the predictive neuroimaging mechanisms of rTMS in patients with MDD. Based on the role of DLPFC and insula in depression, as well as the fact that the left DLPFC is one of the common targets of rTMS, we measured the functional and structural connectivity between the left DLPFC and insula using rs-fMRI and DTI, and analyzed the correlation of connectivity strength with clinical efficacy of rTMS. We found that the functional and structural connectivity between the two regions could positively predict the antidepressant effects; the stronger the connection strength was, the better the clinical efficacy. In addition, we found that only when the position of the left DLPFC was located as a node of CEN, other than located according to previous studies (e.g., the center of BA9 or BA46), the significant positive predictive effects were obtained. These findings provided implications that CEN might be important to rTMS, which could produce therapeutic effects through the connectivity between insula and left DLPFC by directly stimulating the latter region. However, how rTMS takes effects and influences large-scale brain networks through the left DLPFC-insula connectivity is beyond the scope of this article and remains to be investigated in the future.

## Data Availability Statement

The raw data supporting the conclusions of this article will be made available by the authors, without undue reservation.

## Ethics Statement

The studies involving human participants were reviewed and approved by the Local Medical Ethics Committee of The First Affiliated Hospital of Chongqing Medical University. The patients/participants provided their written informed consent to participate in this study. Written informed consent was obtained from the individual(s) for the publication of any potentially identifiable images or data included in this article.

## Author Contributions

LD conceived and designed the experiments. ZX, YX, YC, and XC prepared the samples and performed fMRI for patients. WD and QL performed rTMS in MDD patients. LD, YF, and ZL contributed to the data analysis and wrote the manuscript. All authors contributed to the article and approved the submitted version.

## Conflict of Interest

The authors declare that the research was conducted in the absence of any commercial or financial relationships that could be construed as a potential conflict of interest.

## Footnotes

1 https://www.fil.ion.ucl.ac.uk/spm/

2 http://warwick.ac.uk/snpm

3 https://www.fmrib.ox.ac.uk/fsl

## Supplementary Material

Supplementary Figure 1 When the coordinates of the left DLPFC were located at the center of BA9, **(A)** one sample *t*-test showed positive functional connectivity between left DLPFC and bilateral insula. **(B)** The correlations between the connectivity and the clinical efficacy of rTMS were not significant.

Supplementary Figure 2 When the coordinates of the left DLPFC were located at the center of BA46, **(A)** one sample *t*-test showed positive functional connectivity between left DLPFC and bilateral insula. **(B)** The correlations between the connectivity and the clinical efficacy of rTMS were not significant.
