# Supplementary figures and images for: Functional and Structural Connectivity Between the Left Dorsolateral Prefrontal Cortex and Insula Could Predict the Antidepressant Effects of Repetitive Transcranial Magnetic Stimulation
Source: Front Neurosci. 2021 Mar 26;15:645936. doi: 10.3389/fnins.2021.645936 (PMC8032871; doi:10.3389/fnins.2021.645936)

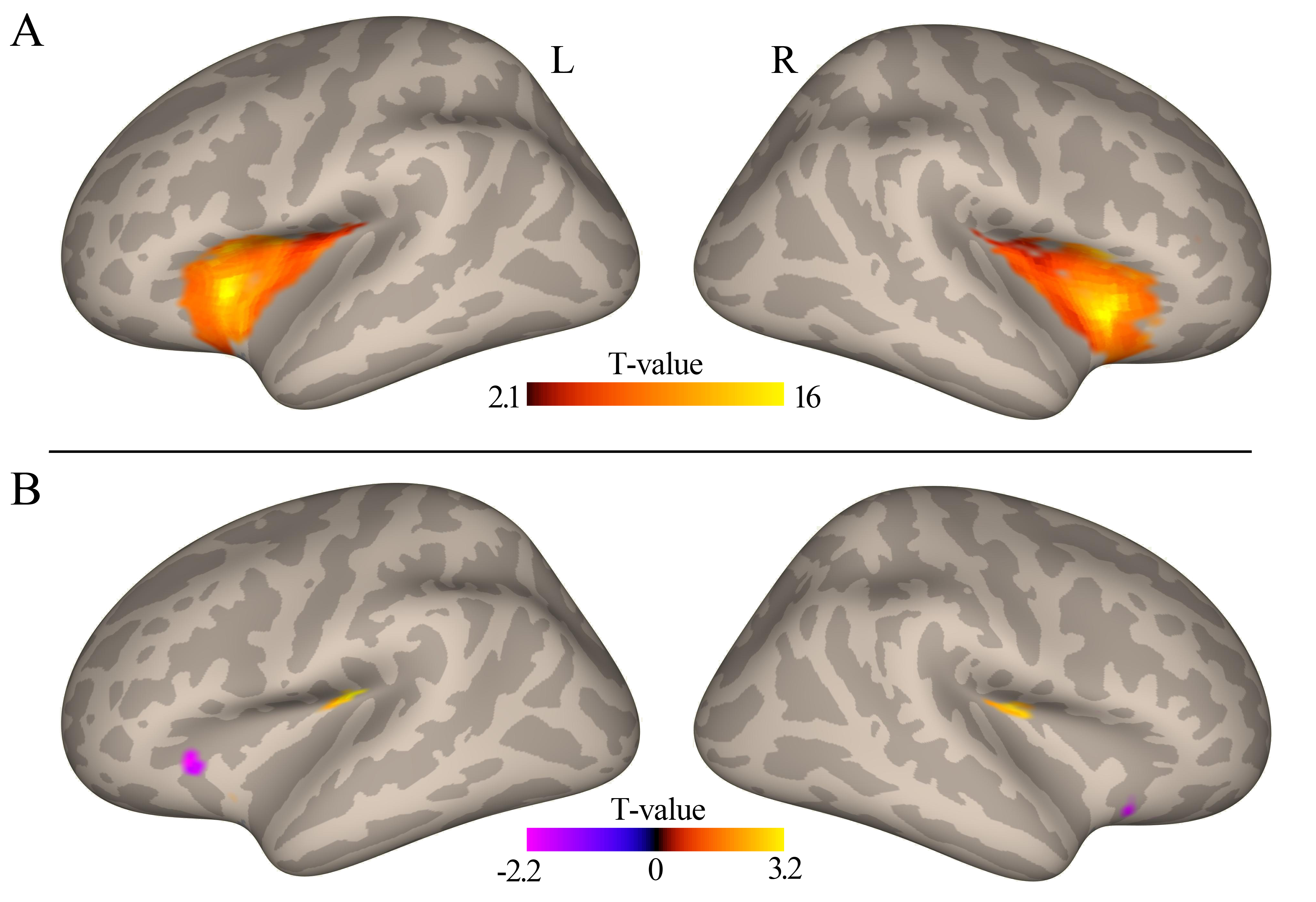

Supplement: Supplementary Figure 1 — When the coordinates of the left DLPFC were located at the center of BA9, (A) one sample t-test showed positive functional connectivity between left DLPFC and bilateral insula. (B) The correlations between the connectivity and the clinical efficacy of rTMS were not significant. [file Image_1.tif]

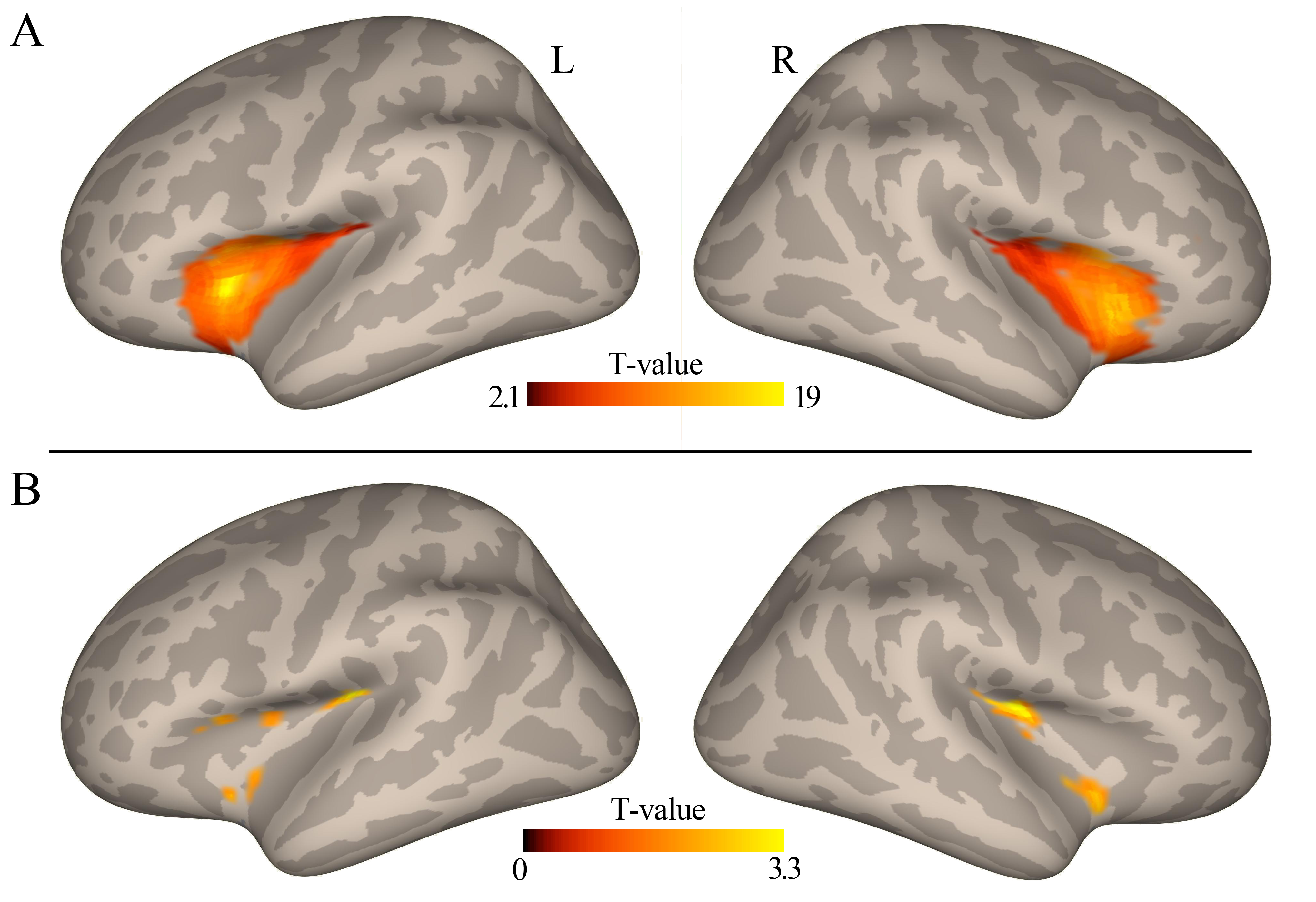

Supplement: Supplementary Figure 2 — When the coordinates of the left DLPFC were located at the center of BA46, (A) one sample t-test showed positive functional connectivity between left DLPFC and bilateral insula. (B) The correlations between the connectivity and the clinical efficacy of rTMS were not significant. [file Image_2.tif]
